# Association between Retinoic *acid receptor-β* hypermethylation and NSCLC risk: a meta-analysis and literature review

**DOI:** 10.18632/oncotarget.14023

**Published:** 2016-12-19

**Authors:** Yan Li, De-guo Lu, Ying-mei Ma, Hongxiang Liu

**Affiliations:** ^1^ Department of Respiratory, The Ninth Peoples Hospital of Chongqing, Chongqing, P. R. China; ^2^ Clinical Laboratory, Linyi Peoples Hospital, Linyi, Shandong, P. R. China; ^3^ Department of Cardiothoracic Surgery, Southwest Hospital, Third Military Medical University, Chongqing, P.R. China

**Keywords:** RARβ, methylation, lung cancer, meta-analysis, tumor suppressor gene

## Abstract

Emerging evidence indicates that Retinoic acid receptor-β (RARβ) is a tumor suppressor in many types of tumor. However, whether or not RARβ is a risk factor and is correlated to clinicopathological characteristics of non-small cell lung cancer (NSCLC) remains unclear. In this report, we performed a meta-analysis to determine the effects of *RARβ* hypermethylation on the incidence of NSCLC and clinicopathological characteristics in human NSCLC patients. Final valuation and analysis of 1780 cancer patients from 16 eligible studies was performed. *RARβ* hypermethylation was found to be significantly higher in NSCLC than in normal lung tissue, the pooled OR from 7 studies including 646 NSCLC and 580 normal lung tissues, OR = 6.05, 95% CI = 3.56-10.25, p<0.00001. *RARβ* hypermethylation was significantly higher in adenocarcinoma (AC) compared to squamous cell carcinoma (SCC), pooled OR is 0.68 (95% CI = 0.52-0.89, p = 0.005). *RARβ* hypermethylation was also found to occur significantly higher in smoker (n = 232) than non-smoker (n = 213) (OR = 2.46, 95% CI = 1.54-3.93, p = 0.0002). Our results indicate that *RARβ* hypermethylation correlates well with an increased risk in NSCLC patients. *RARβ* geneinactivation caused by *RARβ* methylation contributes the NSCLC tumorigenesis and may serve as a potential risk factor, diagnostic marker and drug target of NSCLC.

## INTRODUCTION

Non-small cell lung carcinoma (NSCLC) consists of squamous cell carcinoma (SCC), adenocarcinoma (AC), large cell carcinoma and others. There are approximately 80% of NSCLC cases in later stage where the treatment and prognosis is far from satisfactory [1]. Thus, identification of the risk factors and diagnostic markers is still needed for the prevention and the diagnosis of NSCLC patients. Epigenetic alterations, particularly aberrant DNA methylation, play a crucial role in cancer formation and progression [2–3]. Hypermethylation of CpG island in tumor suppressor gene promoter is a well-known and typical epigenetic changes in cancer [2–4]. Nowadays the assay of gene promoter hypermethylation has not only been recognized as a remarkable tool for diagnosis of cancer, but also a prognosis factor to predict the cancer risk in a variety of cancers including NSCLC [5].

Retinoic acid (RA) and its derivative, retinoid can bind its three retinoic acid receptors (RAR), RARα, RARβ and RARγ, are required for normal lung development [6–9]. Previous reports showed that RARβ was frequently epigenetically silenced in tumor progression, which demonstrated that RARβ belongs to a tumor suppressor protein [10–12]. RARβ gene mutation was not reported in NSCLC. The loss of coactivators, such as AF2 co-activators of the RAR-Thyroid Hormone Receptor complex are often lost in human lung cancer and the loss of AF-2 cofactors results in low levels of transcribed RARβ [13]. Silencing RARβ by promoter hypermethylation has been found as one mechanism that regulates alveolar and epithelial differentiation and lung tumorigenesis [14–15]. The hypermethylation of RARβ in NSCLC has been reported inconsistently [16–17], although some groups indeed found that the inactivation of the RARβ is caused by epigenetic progression, hypermethylation of RARβ gene in NSCLC [16–17]. In addition, it is not clear whether RARβ is associated with any clinical characteristics of NSCLC and predicts the risk of diseases. The discrepancy of the published data underpin the necessity to study the role of RARβ inactivation by gene promoter hypermethylation in NSCLC. Therefore, we perform an evaluation on the role of RARβ hypermethylation in lung tumorigenesis using a quantitative meta-analysis as well as a systematic review.

## RESULTS

**Figure 1:**
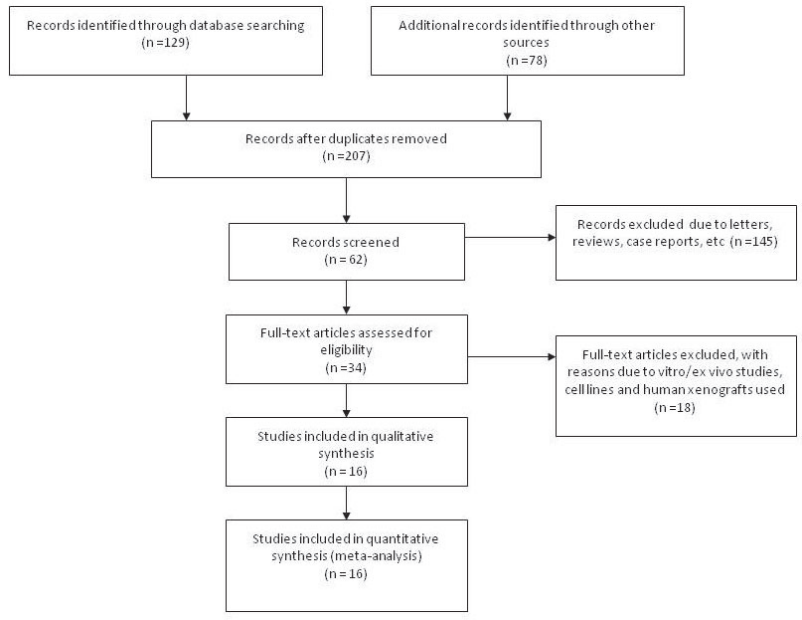
Flow chart of included study

Sixteen publications which fitted into the inclusion criteria were collected into in this study. One hundred and ninety one publications were not included for the reasons such as in vivo animal work or in vitro cell culture model, non-original articles (review and editorial), or irrelevant publications. Figure 1 shows sixteen studies from 2004 to 2014 were included in the current meta-analysis. NSCLC patients from different countries such as China, South Korea, Japan, Finland, Australia, and USA was included and counted 1780 cases. Their demographic information and clinical parameters are formatted into Table 1.

**Table 1:** Clinical features of selected studies

Study	Country	Case No.	Methods	Goal
Li, et al 2014a[49]	China	167	Methylation specific PCR (MSP)	Determine whether tobacco exposure plays a role in gene methylation
Li, et al 2014b[37]	China	56	MSP	Determine the methylation status of three tumor suppressor genes in NSCLC
Zhao, et al 2012[50]	China	80	MSP	Detect methylation of the RARβ gene in tissues from NSCLC patients
Scesnaite, et al 2012[51]	Finland	212	MSP	Determine the methylation status of five tumor suppressor genes in NSCLC
Yanagawa, et al 2011[42]	Japan	62	MSP	Determine the methylation status of five tumor suppressor genes in NSCLC
Zhang, et al 2011[52]	China	200	MSP	Determine the methylation status of three tumor suppressor genes in NSCLC
Liu, et al 2010 [53]	China	80	MSP	Access the methylation status of six tumor suppressor genes in NSCLC
Hawes, et al 2010[54]	USA	117	Methy Light	Determine the DNA methylation status of 27 genes NSCLC
Kubo, et al 2009[55]	Japan	100	MSP	Examine the methylation status in five genes in NSCLC
Seng, et al 2008[56]	Australia	239	MSP	Investigate methylation status of three genes in NSCLC
Hsu, et al 2007[57]	China	82	MSP	Examine 19 genetic and epigenetic markers in NSCLC
Yanagawa, et al 2007[17]	Japan	101	MSP	Determine methylation in 10 genes in NSCLC
Kim, et al 2005a [36]	Korea	72	MSP	Examine the DNA methylation status of five NSCLC
Kim, et al 2005b[58]	Kerea	61	MSP	Examine the methylation status of four tumor suppressor genes in NSCLC
Tomizawa, et al 2004[41]	Japan	120	MSP	Investigate aberrant methylation of three genes in NSCLC
Topaloglu, et al 2004[59]	USA	31	MSP	Examine the methylation status of eight tumor suppressor genes in NSCLC

**Figure 2:**
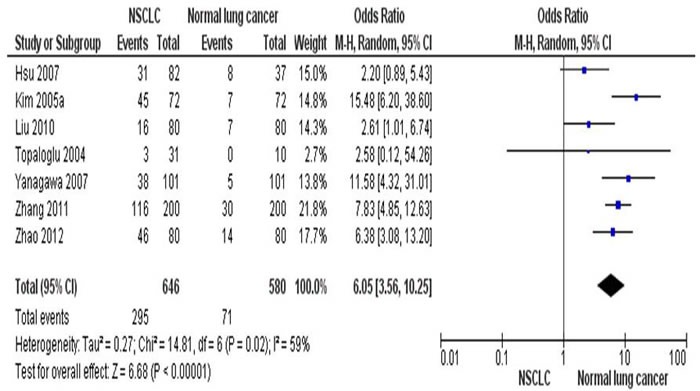
The combining estimates of the odds ratio from 7 selected studies containing lung tissues from 646 of NSCLC patients and 580 of normal individuals is 6.05(95% CI,3.56-10.25, p < 0.00001)

**Figure 3:**
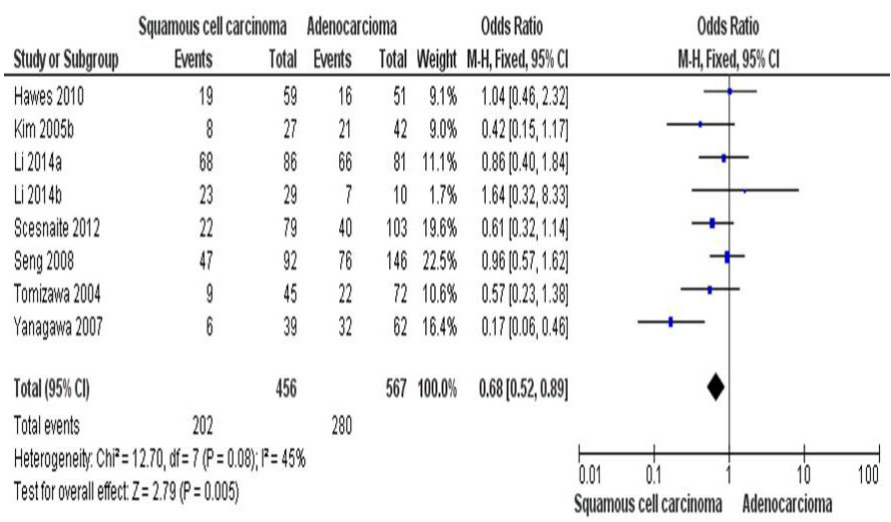
The combining estimates of the odds from 8 studies containing 456 of squamous cell carcinoma (SCC) and 567 of adenocarcinoma (AC) is 0.68 ( 95% C, 0.52-0.89, p = 0.005)

By determine the RARβ promoter hypermethylation using lung tissues, the NSCLC patients showed significantly higher RARβ promter hypermethylation frequency than those of normal individuals. That is the RARβ conveyed the positive association for promoter hypermethylation when comparing NSCLC tumors to normal controls, with the pooled OR of 6.05 (95% CI = 3.56-10.25, p < 0.00001) from 7 studies having both NSCLC tissues (n = 646) and normal lung tissues (n = 580) (Figure 2). To further determine the association of RARβ hypermethylation and two major pathological types, SCC and AC, total of 456 of SCC patients and 567 of AC patients in 8 studies were extracted. RARβ hypermethylation was remarkably higher in AC patients in comparison to SCC patients with pooled OR of 0.68 (95% CI = 0.52-0.89, p = 0.005) (Figure 3). This result demonstrates that the hypermethylation RARβ may have a crucial function in the lung tumorigenesis especially in AC formation.

**Figure 4:**
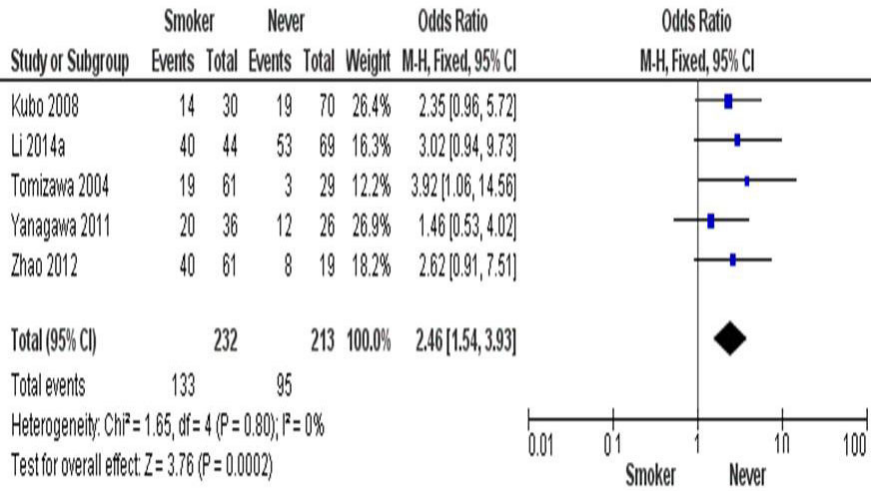
The combining estimates of the odds from 5 studies containing 232 and 213 NSCLC with and without smoking history is 2.46 (95% CI, .54-3.93, p = 0.0002)

After analysis of combining estimates of the OR from 5 studies of 445 of NSCLC (Figure 4), the rate of hypermethylation of RARβ was detected significantly higher in smoker (n = 232) than non-smoker (n = 213) (OR = 2.46, 95% CI = 1.54-3.93, p = 0.0002). This indicates that RARβ conveyed the positive association for promoter hypermethylation when comparing NSCLC smoker and NSCLC non-smoker.

**Figure 5:**
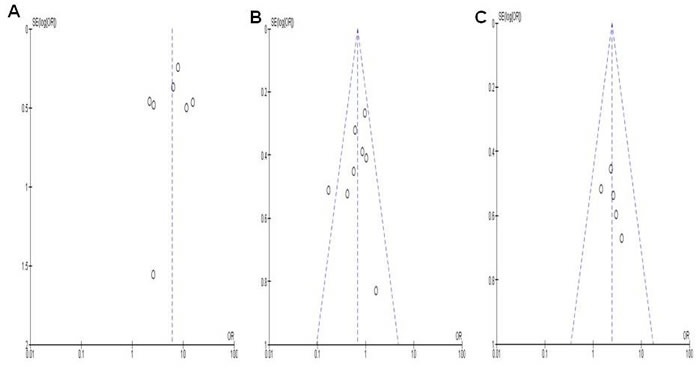
The symmetrical funnel plots demonstrates that no publication biases existed regarding RARβ hypermethylation and clinicopathological features. Panel **A.** showed the funnel plot from 7 studies comparing NSCLC and normal lung tissue. Panel **B.** showed the funnel plot from 8 studies comparing RARβ hypermethylation between squamous cell carcinoma (SCC) and adnocarcinoma (AC). Panel C showed the funnel plot from 5 studies examining the relationship between RARβ hypermethylation and the smokers/ non-smokers in NSCLC patients **C.** X axis: value of Odds ratio (OR); Y axis: Standard errors (SE) multiply log scale of OR.

Finally we removed one study at a time to calculate the sensitivity which is a sign of the analysis result stability. Neither the pooled ORs nor HRs were extremely altered, demonstrating the acceptable stability of the results. The symmetrical funnel plots as shown in Figure 5A-5C imply no publication biases exist in the current study.

## DISCUSSION

DNA methylation is an epigenetic mechanism that carried out by specific enzymes, the DNA methyltransferase enzymes [18–19]. In Eucaryotes, only cytosine belonging to CG dinucleotides, also named CpG in the 5′ position can be methylated, leading to the formation of 5-methylcytosine and are distributed in promoter regions of genes [20–21]. Just similar to other tumor suppressor genes, the CpG islands of RARβ promoter are easily hypermethylated in malignant cells. Gene hypermethylation usually represses its transcription and plays an important role in the gene regulation. The RARβ gene expression can cause RA-dependent and RA-independent apoptosis and growth arrest, which is mediated through RARα [22–23]. RARβ protein results in the expression of a number of its target genes that mediate cell differentiation and death [10, 24–25]. RARβ is epigentically altered in many different kinds of human cancers. Inactivation of RARβ by promoter hypermethylation contributes significantly to tumorigenesis of a variety of cancers including NSCLC [26–32]. Several groups have studied RARβ methylation status in NSCLC; but the lack of systemic and quantitative analysis of methylation of RARβ in NSCLC and its relationship with the clinical parameters really hampers the appreciation of mechanism of lung cancer as well as the development of novel tools for diagnosis and treatment of NSCLC patients. Analysis of the pooled data showed that there was a higher RARβ hypermethylation in NSCLC patients when compared to normal lung tissue, indicating that RARβ hypermethylation is risk factor in the carcinogenesis of NSCLC. RARβ hypermethylation was also found to correlated with pathological types, as well as smoking status. Our findings that NSCLC patients have high frequencies of RARβ gene promoter methylation compared to the normal lung tissues, indicating that the detection of RARβ hypermethylation may provide a practical diagnostic marker for NSCLC patients. Unlike genetic changes, the reversible epigenetic modifications in RARβ gene by methylation can be demethylated and therefore would be pratical to inhibit or delay carcinogenesis. Epigenetically silenced RARβ has been shown to be re-expressed in the presence of histone deacetylase (HDAC) inhibitors and DNA methyltransferase inhibitors (DNMT) inhibitors in RARβ2 silent cancer cells [33]. Treatment with 9-cis RA and an HDAC inhibitor showed effective in a cancer xenografts [34]. Four lung tumor lines treated with 5-aza-2’-deoxycytidine, restored RARβ expression and resulted in decreased tumorigenicity [15]. In addition, curcumin can increase RARβ expression at the mRNA and protein levels in lung cancer A549 and H460 cells, indicating that curcumin is able to inhibit RARβ promoter methylation [35]. The clinical trials have shown the therapeutic potential by reactivation of tumor suppressor expression and may shed light on cancer treatment through gene-targeted therapy.

A number of studies detected and compared the different rate of RARβ hypermethylation; however, the results were contradictory due to limited number of patients from AC and SCC [36–37]. The combining estimates of the odds ratio from 8 publication having 456 SCC and 567 AC is OR = 0.68, 95% CI = 0.52-0.89, p = 0.005, which shows the rate of RARβ hypermethylation was higher in AC than in SCC. In addition, studies have also reported a correlation between DNA methylation and tobacco carcinogens [38–42]. The combining estimates of the odds ratio from 5 studies having 232 and 213 NSCLC with and without smoking history is OR = 2.46, 95% CI = 1.54-3.93, p = 0.0002, shows RARβ hypermethylation significantly increased in smoking NSCLC patients compared to non-smoking NSCLC.

Publication bias did not exist after sensitivity analyses. However the study has several potential restrictions for some reasons. The language was limited to articles published in English. Publications in other languages were exluded due to unavailability of accurate medical translation. In addition, the selection biases could be possibly existed due to observational studies. Cancer cells may silence or repress RARβ by mechanisms other than hypermethylation in NSCLC in order to initiate and promote their growth and resist treatment with RA. Therefore, cautions should be taken when our results are extrapolated to the general populations.

Taken together, our analysis showed a higher RARβ hypermethylation in NSCLC than normal lung tissues, higher in AC than in SCC, and higher in smokers than in non-smokers. RARβ promoter hypermethylation, which inactivated RARβ gene, may significantly contribute to the carcinogenesis and serve as a potential drug target and diagnostic marker for NSCLC patients.

## MATERIALS AND METHODS

### Inclusion and exclusion criteria

The database of Pubmed, Embase, and ISI web of knowledge were searched for publications from May 1, 1998 to December 2015 by the key words: “lung” and “cancer or tumor or neoplasm or carcinoma”, “methylation”, and “RARβ or retinoic acid receptor-β or NR1B2”. In addition, the author bibliographies were also searched manually from the retrieved publications for additional papers.

After withdraw of irrelevant and/or repeated publications from various resources, the remaining papers in the full text were evaluated for selection according to inclusion and exclusion criteria. All searched papers were retrieved. The cited references of selected studies were also determined for other relevant studies. Only one complete study was selected if same patient populations were repeatedly reported.

The inclusion criteria for eligibility include: (1) RARβ hypermethylation was determined in the lung tissues of primary NSCLC patients, (2) RARβ promoter hypermethylation was detected by polymerase chain reaction (PCR), (3) the data provided the information on both status of RARβ hypermethylation as well as clinicopathological indexes of the NSCLC patients (4) the statistic information was given such as individual hazard ratio (HR) of overall survival (OS) and 95 % confidence interval (CI). However, the Letters, reviews, case reports, conference abstracts, editorials, expert opinion, and all research which are involved in vitro/ex vivo studies were not included.

### Extraction methods

We reviewed the following information in each eligible study, authorship, time of publication, sample collection, case numbers, clinicopathological information, the techniques used to calculate gene promoter methylation, methylation frequences and/or gene product expression, and the process of follow-up. Disagreements between the analyzers were fully discussed until come to an agreement. Demographic data and clinical responses were formatted into a table. Heterogeneity of study was calculated to determine if the dataset were suitable for a meta-analysis.

The methodology of each study was evaluated and scored based on the REMARK and ELCWP guidelines [43–44].

### Statistical analysis

Analysis was performed by the softwares of STATA 12.0 (Stata Corporation, TX, USA) and Review Manager 5.2 (Cochrane Collaboration, Oxford, UK). The pooled ratios of RARβ promoter hypermethylation and 95% confidence intervals (95% CI) were calculated. The frequency of RARβ hypermethylation was analyzed and correlated to various tumor phenotypes. Heterogeneity among studies was calculated by Cochran's Q test [45] which showed by the I2[46–47]. When heterogeneity did not exist (I2 values < 50%), a fixed effect model was utilized. On the contrary, a random-effects model was utilized to pool individual data together. In addition, potential sources of heterogeneity were identified by further subgroup analyses. The existence of an association between RARβ promoter hypermethylation and clinicopathological indexes were determined by a pooled OR. P values less than 0.05 was considered significant level.

Publication bias was assessed by using a method reported by Egger et al [48]. We also explored reasons for statistical heterogeneity using meta-regression, subgroup analysis, and sensitivity analysis. The analysis of meta-regression and publication bias were performed by using STATA version 10.0.

## References

[1] Ramalingam S, Belani C (2008). Systemic chemotherapy for advanced non-small cell lung cancer: recent advances and future directions. Oncologist.

[2] Delpu Y, Cordelier P, Cho WC, Torrisani J (2013). DNA methylation and cancer diagnosis. Int J Mol Sci.

[3] Ma X, Wang YW, Zhang MQ, Gazdar AF (2013). DNA methylation data analysis and its application to cancer research. Epigenomics.

[4] Ghavifekr Fakhr M, Farshdousti Hagh M, Shanehbandi D, Baradaran B (2013). DNA Methylation Pattern as Important Epigenetic Criterion in Cancer. Genet Res Int.

[5] Fleischhacker M, Dietrich D, Liebenberg V, Field JK, Schmidt B (2013). The role of DNA methylation as biomarkers in the clinical management of lung cancer. Expert Rev Respir Med.

[6] Grummer MA, Thet LA, Zachman RD (1994). Expression of retinoic acid receptor genes in fetal and newborn rat lung. Pediatr Pulmonol.

[7] Mendelsohn C, Lohnes D, Decimo D, Lufkin T, LeMeur M, Chambon P, Mark M (1994). Function of the retinoic acid receptors (RARs) during development (II). Multiple abnormalities at various stages of organogenesis in RAR double mutants. Development.

[8] Chytil F (1996). Retinoids in lung development. FASEB J.

[9] Minna JD, Mangelsdorf DJ (1997). Retinoic acid receptor expression abnormalities in lung cancer: important clues or major obstacles?. J Natl Cancer Inst.

[10] Alvarez S, Germain P, Alvarez R, Rodriguez-Barrios F, Gronemeyer H, de LAR (2007). Structure, function and modulation of retinoic acid receptor beta, a tumor suppressor. Int J Biochem Cell Biol.

[11] Mongan NP, Gudas LJ (2007). Diverse actions of retinoid receptors in cancer prevention and treatment. Differentiation.

[12] Swift CB, Hays JL, Petty WJ (2008). Distinct functions of retinoic acid receptor beta isoforms: implications for targeted therapy. Endocr Metab Immune Disord Drug Targets.

[13] Moghal N, Neel BG (1995). Evidence for impaired retinoic acid receptor-thyroid hormone receptor AF-2 cofactor activity in human lung cancer. Mol Cell Biol.

[14] Licchesi JD, Westra WH, Hooker CM, Herman JG (2008). Promoter hypermethylation of hallmark cancer genes in atypical adenomatous hyperplasia of the lung. Clin Cancer Res.

[15] Virmani AK, Rathi A, Zochbauer-Muller S, Sacchi N, Fukuyama Y, Bryant D, Maitra A, Heda S, Fong KM, Thunnissen F, Minna JD, Gazdar AF (2000). Promoter methylation and silencing of the retinoic acid receptor-beta gene in lung carcinomas. J Natl Cancer Inst.

[16] Umemura S, Fujimoto N, Hiraki A, Gemba K, Takigawa N, Fujiwara K, Fujii M, Umemura H, Satoh M, Tabata M, Ueoka H, Kiura K, Kishimoto T, Tanimoto M (2008). Aberrant promoter hypermethylation in serum DNA from patients with silicosis. Carcinogenesis.

[17] Yanagawa N, Tamura G, Oizumi H, Kanauchi N, Endoh M, Sadahiro M, Motoyama T (2007). Promoter hypermethylation of RASSF1A and RUNX3 genes as an independent prognostic prediction marker in surgically resected non-small cell lung cancers. Lung Cancer.

[18] Bestor TH (2000). Gene silencing as a threat to the success of gene therapy. J Clin Invest.

[19] Hamidi T, Singh AK, Chen T (2015). Genetic alterations of DNA methylation machinery in human diseases. Epigenomics.

[20] Kulis M, Queiros AC, Beekman R, Martin-Subero JI (2013). Intragenic DNA methylation in transcriptional regulation, normal differentiation and cancer. Biochim Biophys Acta.

[21] Klutstein M, Nejman D, Greenfield R, Cedar H (2016). DNA Methylation in Cancer and Aging. Cancer Res.

[22] Liu Y, Lee MO, Wang HG, Li Y, Hashimoto Y, Klaus M, Reed JC, Zhang X (1996). Retinoic acid receptor beta mediates the growth-inhibitory effect of retinoic acid by promoting apoptosis in human breast cancer cells. Mol Cell Biol.

[23] Chambon P (1996). A decade of molecular biology of retinoic acid receptors. FASEB J.

[24] Bushue N, Wan YJ (2010). Retinoid pathway and cancer therapeutics. Adv Drug Deliv Rev.

[25] Tang XH, Gudas LJ (2011). Retinoids, retinoic acid receptors, and cancer. Annu Rev Pathol.

[26] Hua F, Fang N, Li X, Zhu S, Zhang W, Gu J (2014). A meta-analysis of the relationship between RARbeta gene promoter methylation and non-small cell lung cancer. Plos one.

[27] Chen R, Ren S, Meng T, Aguilar J, Sun Y (2013). Impact of glutathione-S-transferases (GST) polymorphisms and hypermethylation of relevant genes on risk of prostate cancer biochemical recurrence: a meta-analysis. Plos one.

[28] Moison C, Assemat F, Daunay A, Tost J, Guieysse-Peugeot AL, Arimondo PB (2014). Synergistic chromatin repression of the tumor suppressor gene RARB in human prostate cancers. Epigenetics.

[29] Li W, Deng J, Jiang P, Zeng X, Hu S, Tang J (2012). Methylation of the RASSF1A and RARbeta genes as a candidate biomarker for lung cancer. Exp Ther Med.

[30] Piperi C, Themistocleous MS, Papavassiliou GA, Farmaki E, Levidou G, Korkolopoulou P, Adamopoulos C, Papavassiliou AG (2010). High incidence of MGMT and RARbeta promoter methylation in primary glioblastomas: association with histopathological characteristics, inflammatory mediators and clinical outcome. Mol Med.

[31] Shukla S, Mirza S, Sharma G, Parshad R, Gupta SD, Ralhan R (2006). Detection of RASSF1A and RARbeta hypermethylation in serum DNA from breast cancer patients. Epigenetics.

[32] Fischer JR, Ohnmacht U, Rieger N, Zemaitis M, Stoffregen C, Kostrzewa M, Buchholz E, Manegold C, Lahm H (2006). Promoter methylation of RASSF1A, RARbeta and DAPK predict poor prognosis of patients with malignant mesothelioma. Lung Cancer.

[33] Sirchia SM, Ren M, Pili R, Sironi E, Somenzi G, Ghidoni R, Toma S, Nicolo G, Sacchi N (2002). Endogenous reactivation of the RARbeta2 tumor suppressor gene epigenetically silenced in breast cancer. Cancer Res.

[34] Qian DZ, Ren M, Wei Y, Wang X, van dGF Rasmussen C, Nakanishi O, Sacchi N, Pili R In (2005). vivo imaging of retinoic acid receptor beta2 transcriptional activation by the histone deacetylase inhibitor MS-275 in retinoid-resistant prostate cancer cells. Prostate.

[35] Jiang A, Wang X, Shan X, Li Y, Wang P, Jiang P, Feng Q (2015). Curcumin Reactivates Silenced Tumor Suppressor Gene RARbeta by Reducing DNA Methylation. Phytother Res.

[36] Kim YT, Park SJ, Lee SH, Kang HJ, Hahn S, Kang CH, Sung SW, Kim JH (2005). Prognostic implication of aberrant promoter hypermethylation of CpG islands in adenocarcinoma of the lung. J Thorac Cardiovasc Surg.

[37] Li W, Deng J, Tang JX (2014). Combined effects methylation of FHIT, RASSF1A and RARbeta genes on non-small cell lung cancer in the Chinese population. Asian Pac J Cancer Prev.

[38] Lee YW, Klein CB, Kargacin B, Salnikow K, Kitahara J, Dowjat K, Zhitkovich A, Christie NT, Costa M (1995). Carcinogenic nickel silences gene expression by chromatin condensation and DNA methylation: a new model for epigenetic carcinogens. Mol Cell Biol.

[39] Swafford DS, Middleton SK, Palmisano WA, Nikula KJ, Tesfaigzi J, Baylin SB, Herman JG, Belinsky SA (1997). Frequent aberrant methylation of p16INK4a in primary rat lung tumors. Mol Cell Biol.

[40] Rom WN, Hay JG, Lee TC, Jiang Y, Tchou-Wong KM (2000). Molecular and genetic aspects of lung cancer. Am J Respir Crit Care Med.

[41] Tomizawa Y, Iijima H, Nomoto T, Iwasaki Y, Otani Y, Tsuchiya S, Saito R, Dobashi K, Nakajima T, Mori M (2004). Clinicopathological significance of aberrant methylation of RARbeta2 at 3p24, RASSF1A at 3p21.3, and FHIT at 3p14.2 in patients with non-small cell lung cancer. Lung Cancer.

[42] Yanagawa N, Tamura G, Oizumi H, Endoh M, Sadahiro M, Motoyama T (2011). Inverse correlation between EGFR mutation and FHIT, RASSF1A and RUNX3 methylation in lung adenocarcinoma: relation with smoking status. Anticancer Res.

[43] McShane LM, Altman DG, Sauerbrei W, Taube SE, Gion M, Clark GM (2005). Reporting recommendations for tumor marker prognostic studies (REMARK). J Natl Cancer Inst.

[44] Steels E, Paesmans M, Berghmans T, Branle F, Lemaitre F, Mascaux C, Meert AP, Vallot F, Lafitte JJ, Sculier JP (2001). Role of p53 as a prognostic factor for survival in lung cancer: a systematic review of the literature with a meta-analysis. Eur Respir J.

[45] DerSimonian R, Laird N (1986). Meta-analysis in clinical trials. Control Clin Trials.

[46] Higgins JP, Thompson SG, Deeks JJ, Altman DG (2003). Measuring inconsistency in meta-analyses. BMJ.

[47] DerSimonian R (1996). Meta-analysis in the design and monitoring of clinical trials. Stat Med.

[48] Egger M, Davey Smith G, Schneider M, Minder C (1997). Bias in meta-analysis detected by a simple, graphical test. BMJ.

[49] Li W, Deng J, Wang SS, Ma L, Pei J, Zeng XX, Tang JX (2014). Association of methylation of the RAR-beta gene with cigarette smoking in non-small cell lung cancer with Southern-Central Chinese population. Asian Pac J Cancer Prev.

[50] Zhao X, Wang N, Zhang M, Xue S, Shi K, Chen Z (2012). Detection of methylation of the RAR-beta gene in patients with non-small cell lung cancer. Oncol Lett.

[51] Scesnaite A, Jarmalaite S, Mutanen P, Anttila S, Nyberg F, Benhamou S, Boffetta P, Husgafvel-Pursiainen K (2012). Similar DNA methylation pattern in lung tumours from smokers and never-smokers with second-hand tobacco smoke exposure. Mutagenesis.

[52] Zhang CY, Jin YT, Xu HY, Zhang H, Zhang WM, Sun XY, Tan C, Chen CM (2011). [Relationship between promoter methylation of p16, DAPK and RAR beta genes and the clinical data of non-small cell lung cancer]. Zhonghua Yi Xue Yi Chuan Xue Za Zhi.

[53] Liu Z, Li W, Lei Z, Zhao J, Chen XF, Liu R, Peng X, Wu ZH, Chen J, Liu H, Zhou QH, Zhang HT (2010). CpG island methylator phenotype involving chromosome 3p confers an increased risk of non-small cell lung cancer. J Thorac Oncol.

[54] Hawes SE, Stern JE, Feng Q, Wiens LW, Rasey JS, Lu H, Kiviat NB, Vesselle H (2010). DNA hypermethylation of tumors from non-small cell lung cancer (NSCLC) patients is associated with gender and histologic type. Lung Cancer.

[55] Kubo T, Yamamoto H, Ichimura K, Jida M, Hayashi T, Otani H, Tsukuda K, Sano Y, Kiura K, Toyooka S (2009). DNA methylation in small lung adenocarcinoma with bronchioloalveolar carcinoma components. Lung Cancer.

[56] Seng TJ, Currey N, Cooper WA, Lee CS, Chan C, Horvath L, Sutherland RL, Kennedy C, McCaughan B, Kohonen-Corish MR (2008). DLEC1 and MLH1 promoter methylation are associated with poor prognosis in non-small cell lung carcinoma. Br J Cancer.

[57] Hsu HS, Chen TP, Wen CK, Hung CH, Chen CY, Chen JT, Wang YC (2007). Multiple genetic and epigenetic biomarkers for lung cancer detection in cytologically negative sputum and a nested case-control study for risk assessment. J Pathol.

[58] Kim YT, Lee SH, Sung SW, Kim JH (2005). Can aberrant promoter hypermethylation of CpG islands predict the clinical outcome of non-small cell lung cancer after curative resection?. Ann Thorac Surg.

[59] Topaloglu O, Hoque MO, Tokumaru Y, Lee J, Ratovitski E, Sidransky D, Moon CS (2004). Detection of promoter hypermethylation of multiple genes in the tumor and bronchoalveolar lavage of patients with lung cancer. Clin Cancer Res.

